# 7-Nitro-1,2,3,4-tetra­hydro­naphthalene-1-spiro-2′-(1,3-dithiane)

**DOI:** 10.1107/S1600536809001536

**Published:** 2009-01-17

**Authors:** Hoong-Kun Fun, Reza Kia, Annada C. Maity, Shyamaprosad Goswami

**Affiliations:** a X-ray Crystallography Unit, School of Physics, Universiti Sains Malaysia, 11800 USM, Penang, Malaysia; b Department of Chemistry, Bengal Engineering and Science University, Shibpur, Howrah 711 103, India

## Abstract

In the title compound, C_13_H_15_NO_2_S_2_, the nitro group is coplanar with the benzene ring to which it is attached, forming a dihedral angle of 1.07 (14)°. The dithiane ring adopts a chair conformation. In the crystal structure, mol­ecules are linked through C—H⋯O and C—H⋯π [C⋯*Cg* = 3.7164 (15) Å] inter­actions. The crystal studied was an inversion twin with an 0.134 (5):0.866 (5) domain ratio.

## Related literature

For the calculation of ring puckering parameters, see: Cremer & Pople (1975 ▶). For related literature including applications, see: Goswami & Maity (2008 ▶); Fun *et al.* (2009 ▶).
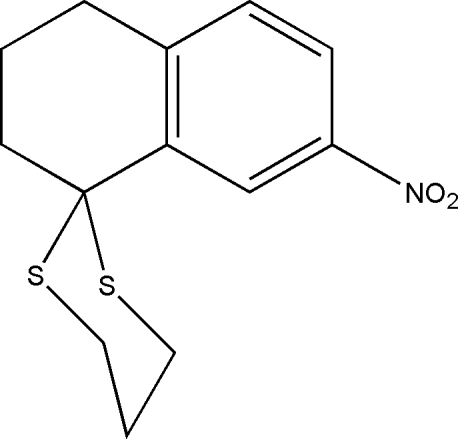


## Experimental

### 

#### Crystal data

C_13_H_15_NO_2_S_2_
*M* *_r_* = 281.38
Orthorhombic, 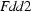

*a* = 12.8673 (1) Å
*b* = 42.2330 (6) Å
*c* = 9.1819 (1) Å
*V* = 4989.67 (10) Å^3^
*Z* = 16
Mo *K*α radiation
μ = 0.42 mm^−1^
*T* = 100.0 (1) K
0.41 × 0.30 × 0.06 mm

#### Data collection

Bruker SMART APEXII CCD area-detector diffractometer
Absorption correction: multi-scan (**SADABS**; Bruker, 2005 ▶) *T*_min_ = 0.849, *T*_max_ = 0.977
54206 measured reflections
6726 independent reflections
5979 reflections with *I* > 2σ(*I*)
*R*_int_ = 0.067

#### Refinement

*R*[*F* ^2^ > 2σ(*F* ^2^)] = 0.042
*wR*(*F* ^2^) = 0.077
*S* = 1.06
6726 reflections
164 parameters
1 restraint
H-atom parameters constrained
Δρ_max_ = 0.42 e Å^−3^
Δρ_min_ = −0.23 e Å^−3^
Absolute structure: Flack (1983 ▶), 3202 Friedel pairs
Flack parameter: 0.13 (5)

### 

Data collection: *APEX2* (Bruker, 2005 ▶); cell refinement: *SAINT* (Bruker, 2005 ▶); data reduction: *SAINT*; program(s) used to solve structure: *SHELXTL* (Sheldrick, 2008 ▶); program(s) used to refine structure: *SHELXTL*; molecular graphics: *SHELXTL*; software used to prepare material for publication: *SHELXTL* and *PLATON* (Spek, 2003 ▶).

## Supplementary Material

Crystal structure: contains datablocks global, I. DOI: 10.1107/S1600536809001536/tk2351sup1.cif

Structure factors: contains datablocks I. DOI: 10.1107/S1600536809001536/tk2351Isup2.hkl

Additional supplementary materials:  crystallographic information; 3D view; checkCIF report

## Figures and Tables

**Table 1:** Hydrogen-bond geometry (Å, °)

*D*—H⋯*A*	*D*—H	H⋯*A*	*D*⋯*A*	*D*—H⋯*A*
C13—H13*A*⋯O1^i^	0.97	2.58	3.4565 (18)	151
C7—H7*A*⋯*Cg*1^ii^	0.93	2.82	3.7164 (15)	162

Symmetry codes: (i) 
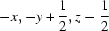
; (ii) 
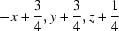
. *Cg*1 is the centroid of the C5–C10 benzene ring.

**Fig. 1.**
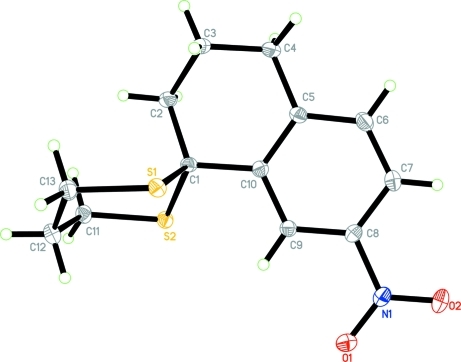
The molecular structure of (I), showing 50% probability displacement ellipsoids and the atomic numbering.

**Fig. 2.**
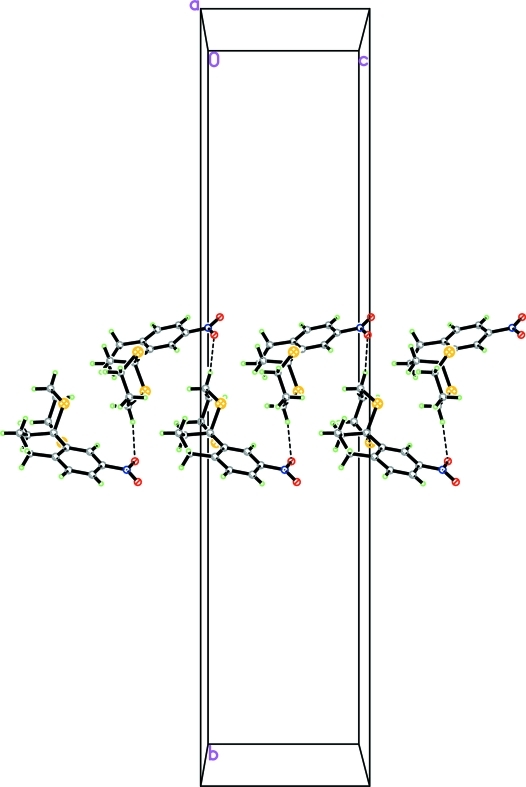
The crystal packing of (I), viewed down the a-axis showing the linking of molecules through intermolecular C—H···O interactions (dashed lines) along the c-axis.

## supplementary crystallographic information

### Comment

The protection of carbonyl groups to produce dithioketals is now commonly used as an important synthetic technique in the preparation of many organic compounds, including multi-functional complex molecules (Goswami & Maity 2008; Fun *et al.*, 2009). Herein, we report the synthesis of the title compound, (I), from 5-nitro-3,4-dihydro-2H-naphthalen-1-one using boron trifluoride etherate as the catalyst.

In (I), Fig. 1, the nitro group is co-planar with the benzene ring, forming a dihedral angle of 1.07 (14)°. The thiacyclohexane ring adopts a chair conformation with ring puckering parameters of Q = 0.7198 (11) Å, Θ = 8.49 (10)°, and Φ = 79.6 (6)° (Cremer & Pople, 1975). The crystal structure is stabilized by intermolecular C—H···π interactions (*Cg*1 is the centroid of the C5–C10 benzene ring), see Table 1. Further, neighbouring molecules are linked through C—H···O interactions along the *c* axis, Fig. 2.

### Experimental

A stirred dichloromethane (50 mL) solution of 5-nitro-3,4-dihydro-2H-naphthalen-1-one (500 mg, 2.61 mmol) and boron trifluoride etherate (0.5 mL) in was cooled to 273 K. To this 1,3-propanedithiol (450 mg, 4.1 mmol) was added dropwise over 15 min. The mixture was stirred at room temperature for 3 h and the progress of the reaction was monitored by TLC. After completion of the reaction, NaHCO_3_ solution was added carefully to neutralize the mixture at room temperature. This was then extracted with dichloromethane. The organic layer was dried (anhydrous Na_2_SO_4_) and the solvent removed under reduced pressure. The crude product was purified by column chromatography using silica gel with 10 % ethyl acetate in petroleum ether as eluant to afford (I) (670 mg, 92 %) as a colourless crystalline solid along with other thiane derivatives.

### Refinement

All hydrogen atoms were positioned geometrically and refined in the riding model approximation with C—H = 0.93-0.97 Å, and with U_iso_(H) = 1.2 U_eq_(C). The structure was twinned with a refined BASF ratio of 0.134 (5):0.866 (5).

### Crystal data

**Table:**

C_13_H_15_NO_2_S_2_	*F*(000) = 2368
*M**_r_* = 281.38	*D*_x_ = 1.498 Mg m^−^^3^
Orthorhombic, *F**d**d*2	Mo *K*α radiation, λ = 0.71073 Å
Hall symbol: F 2 -2d	Cell parameters from 9946 reflections
*a* = 12.8673 (1) Å	θ = 2.8–35.0°
*b* = 42.2330 (6) Å	µ = 0.42 mm^−^^1^
*c* = 9.1819 (1) Å	*T* = 100 K
*V* = 4989.67 (10) Å^3^	Plate, colourless
*Z* = 16	0.41 × 0.30 × 0.06 mm

### Data collection

**Table:**

Bruker SMART APEXII CCD area-detector diffractometer	6726 independent reflections
Radiation source: fine-focus sealed tube	5979 reflections with *I* > 2σ(*I*)
graphite	*R*_int_ = 0.067
φ and ω scans	θ_max_ = 37.9°, θ_min_ = 1.9°
Absorption correction: multi-scan (*SADABS*; Bruker, 2005)	*h* = −22→22
*T*_min_ = 0.849, *T*_max_ = 0.977	*k* = −72→72
54206 measured reflections	*l* = −15→15

### Refinement

**Table:**

Refinement on *F*^2^	Secondary atom site location: difference Fourier map
Least-squares matrix: full	Hydrogen site location: inferred from neighbouring sites
*R*[*F*^2^ > 2σ(*F*^2^)] = 0.042	H-atom parameters constrained
*wR*(*F*^2^) = 0.077	*w* = 1/[σ^2^(*F*_o_^2^) + (0.0258*P*)^2^ + 6.6616*P*] where *P* = (*F*_o_^2^ + 2*F*_c_^2^)/3
*S* = 1.06	(Δ/σ)_max_ = 0.001
6726 reflections	Δρ_max_ = 0.42 e Å^−^^3^
164 parameters	Δρ_min_ = −0.23 e Å^−^^3^
1 restraint	Absolute structure: Flack (1983), 3202 Friedel pairs
Primary atom site location: structure-invariant direct methods	Flack parameter: 0.13 (5)

### Special details

**Table:**

Experimental. The low-temperature data was collected with the Oxford Cyrosystem Cobra low-temperature attachment.
Geometry. All esds (except the esd in the dihedral angle between two l.s. planes) are estimated using the full covariance matrix. The cell esds are taken into account individually in the estimation of esds in distances, angles and torsion angles; correlations between esds in cell parameters are only used when they are defined by crystal symmetry. An approximate (isotropic) treatment of cell esds is used for estimating esds involving l.s. planes.
Refinement. Refinement of F^2^ against ALL reflections. The weighted R-factor wR and goodness of fit S are based on F^2^, conventional R-factors R are based on F, with F set to zero for negative F^2^. The threshold expression of F^2^ > 2sigma(F^2^) is used only for calculating R-factors(gt) etc. and is not relevant to the choice of reflections for refinement. R-factors based on F^2^ are statistically about twice as large as those based on F, and R- factors based on ALL data will be even larger.

### Fractional atomic coordinates and isotropic or equivalent isotropic displacement parameters (Å^2^)

**Table:**

	*x*	*y*	*z*	*U*_iso_*/*U*_eq_	
S1	0.10407 (2)	0.241432 (7)	0.34587 (3)	0.01374 (6)	
S2	−0.04426 (2)	0.186537 (7)	0.31249 (4)	0.01436 (6)	
O1	0.08674 (9)	0.16135 (3)	0.79752 (12)	0.0220 (2)	
O2	0.22833 (9)	0.13439 (3)	0.82907 (13)	0.0258 (2)	
N1	0.16994 (9)	0.15045 (3)	0.75344 (12)	0.0163 (2)	
C1	0.08854 (9)	0.20066 (3)	0.27797 (13)	0.01231 (19)	
C2	0.11450 (10)	0.19792 (3)	0.11508 (14)	0.0159 (2)	
H2A	0.0911	0.1775	0.0791	0.019*	
H2B	0.0776	0.2143	0.0618	0.019*	
C3	0.23059 (10)	0.20125 (3)	0.08744 (15)	0.0167 (2)	
H3A	0.2552	0.2212	0.1269	0.020*	
H3B	0.2440	0.2011	−0.0165	0.020*	
C4	0.28806 (11)	0.17388 (3)	0.15959 (15)	0.0167 (2)	
H4A	0.2745	0.1546	0.1055	0.020*	
H4B	0.3622	0.1779	0.1556	0.020*	
C5	0.25624 (9)	0.16909 (3)	0.31606 (15)	0.0139 (2)	
C6	0.32137 (11)	0.15154 (3)	0.40812 (16)	0.0173 (2)	
H6A	0.3840	0.1439	0.3719	0.021*	
C7	0.29507 (11)	0.14526 (3)	0.55123 (16)	0.0169 (2)	
H7A	0.3388	0.1335	0.6112	0.020*	
C8	0.20103 (10)	0.15708 (3)	0.60257 (14)	0.0140 (2)	
C9	0.13528 (10)	0.17506 (3)	0.51660 (14)	0.0132 (2)	
H9A	0.0739	0.1831	0.5550	0.016*	
C10	0.16192 (10)	0.18097 (3)	0.37155 (13)	0.0127 (2)	
C11	−0.11830 (10)	0.21555 (3)	0.20993 (15)	0.0160 (2)	
H11A	−0.0973	0.2145	0.1086	0.019*	
H11B	−0.1913	0.2100	0.2147	0.019*	
C12	−0.10538 (11)	0.24943 (3)	0.26325 (16)	0.0176 (2)	
H12A	−0.1238	0.2503	0.3656	0.021*	
H12B	−0.1535	0.2629	0.2107	0.021*	
C13	0.00429 (10)	0.26252 (3)	0.24439 (15)	0.0164 (2)	
H13A	0.0046	0.2845	0.2747	0.020*	
H13B	0.0220	0.2619	0.1418	0.020*	

### Atomic displacement parameters (Å^2^)

**Table:**

	*U*^11^	*U*^22^	*U*^33^	*U*^12^	*U*^13^	*U*^23^
S1	0.01297 (12)	0.01371 (11)	0.01454 (13)	0.00002 (10)	−0.00051 (10)	−0.00104 (10)
S2	0.01150 (11)	0.01563 (11)	0.01593 (13)	−0.00058 (10)	−0.00027 (10)	0.00191 (10)
O1	0.0251 (5)	0.0258 (5)	0.0152 (4)	0.0057 (4)	0.0027 (4)	−0.0002 (4)
O2	0.0277 (5)	0.0308 (5)	0.0188 (5)	0.0073 (4)	−0.0041 (4)	0.0086 (4)
N1	0.0198 (5)	0.0157 (4)	0.0135 (5)	0.0000 (4)	−0.0024 (4)	0.0006 (4)
C1	0.0110 (5)	0.0143 (4)	0.0117 (5)	−0.0003 (4)	0.0009 (4)	−0.0003 (4)
C2	0.0159 (5)	0.0197 (5)	0.0121 (5)	0.0014 (4)	0.0012 (4)	−0.0010 (4)
C3	0.0171 (5)	0.0185 (5)	0.0146 (5)	0.0009 (4)	0.0045 (4)	0.0010 (4)
C4	0.0154 (5)	0.0187 (5)	0.0159 (5)	0.0023 (4)	0.0047 (4)	0.0002 (4)
C5	0.0123 (5)	0.0135 (4)	0.0158 (5)	−0.0003 (4)	0.0018 (4)	−0.0002 (4)
C6	0.0141 (5)	0.0172 (5)	0.0205 (6)	0.0031 (4)	0.0015 (5)	0.0006 (5)
C7	0.0153 (5)	0.0156 (5)	0.0197 (6)	0.0020 (4)	−0.0024 (4)	0.0013 (4)
C8	0.0158 (5)	0.0137 (4)	0.0124 (5)	−0.0012 (4)	−0.0010 (4)	0.0003 (4)
C9	0.0123 (5)	0.0146 (5)	0.0128 (5)	−0.0001 (4)	−0.0003 (4)	−0.0006 (4)
C10	0.0116 (5)	0.0135 (4)	0.0129 (5)	−0.0009 (4)	−0.0002 (4)	−0.0002 (4)
C11	0.0129 (5)	0.0179 (5)	0.0173 (6)	0.0011 (4)	−0.0022 (4)	0.0010 (4)
C12	0.0158 (6)	0.0164 (5)	0.0207 (6)	0.0033 (4)	−0.0006 (5)	−0.0002 (4)
C13	0.0152 (5)	0.0152 (5)	0.0187 (6)	0.0019 (4)	−0.0006 (4)	0.0013 (4)

### Geometric parameters (Å, °)

**Table:**

S1—C13	1.8192 (13)	C5—C6	1.4022 (18)
S1—C1	1.8421 (12)	C5—C10	1.4086 (17)
S2—C11	1.8153 (13)	C6—C7	1.383 (2)
S2—C1	1.8375 (12)	C6—H6A	0.9300
O1—N1	1.2337 (16)	C7—C8	1.3915 (18)
O2—N1	1.2276 (15)	C7—H7A	0.9300
N1—C8	1.4688 (17)	C8—C9	1.3840 (17)
C1—C10	1.5236 (17)	C9—C10	1.3977 (17)
C1—C2	1.5369 (17)	C9—H9A	0.9300
C2—C3	1.5217 (18)	C11—C12	1.5216 (19)
C2—H2A	0.9700	C11—H11A	0.9700
C2—H2B	0.9700	C11—H11B	0.9700
C3—C4	1.5237 (18)	C12—C13	1.5254 (19)
C3—H3A	0.9700	C12—H12A	0.9700
C3—H3B	0.9700	C12—H12B	0.9700
C4—C5	1.5075 (19)	C13—H13A	0.9700
C4—H4A	0.9700	C13—H13B	0.9700
C4—H4B	0.9700		
			
C13—S1—C1	101.98 (6)	C7—C6—H6A	119.1
C11—S2—C1	100.34 (6)	C5—C6—H6A	119.1
O2—N1—O1	123.49 (12)	C6—C7—C8	117.77 (12)
O2—N1—C8	118.18 (11)	C6—C7—H7A	121.1
O1—N1—C8	118.33 (11)	C8—C7—H7A	121.1
C10—C1—C2	111.90 (10)	C9—C8—C7	122.36 (12)
C10—C1—S2	107.56 (8)	C9—C8—N1	118.43 (11)
C2—C1—S2	110.21 (9)	C7—C8—N1	119.20 (11)
C10—C1—S1	104.60 (8)	C8—C9—C10	119.44 (11)
C2—C1—S1	112.11 (8)	C8—C9—H9A	120.3
S2—C1—S1	110.24 (6)	C10—C9—H9A	120.3
C3—C2—C1	111.63 (11)	C9—C10—C5	119.50 (11)
C3—C2—H2A	109.3	C9—C10—C1	118.87 (11)
C1—C2—H2A	109.3	C5—C10—C1	121.62 (11)
C3—C2—H2B	109.3	C12—C11—S2	114.24 (9)
C1—C2—H2B	109.3	C12—C11—H11A	108.7
H2A—C2—H2B	108.0	S2—C11—H11A	108.7
C2—C3—C4	109.49 (11)	C12—C11—H11B	108.7
C2—C3—H3A	109.8	S2—C11—H11B	108.7
C4—C3—H3A	109.8	H11A—C11—H11B	107.6
C2—C3—H3B	109.8	C11—C12—C13	113.91 (11)
C4—C3—H3B	109.8	C11—C12—H12A	108.8
H3A—C3—H3B	108.2	C13—C12—H12A	108.8
C5—C4—C3	112.60 (11)	C11—C12—H12B	108.8
C5—C4—H4A	109.1	C13—C12—H12B	108.8
C3—C4—H4A	109.1	H12A—C12—H12B	107.7
C5—C4—H4B	109.1	C12—C13—S1	114.67 (9)
C3—C4—H4B	109.1	C12—C13—H13A	108.6
H4A—C4—H4B	107.8	S1—C13—H13A	108.6
C6—C5—C10	119.02 (12)	C12—C13—H13B	108.6
C6—C5—C4	118.89 (11)	S1—C13—H13B	108.6
C10—C5—C4	122.07 (11)	H13A—C13—H13B	107.6
C7—C6—C5	121.88 (12)		
			
C11—S2—C1—C10	173.57 (8)	O2—N1—C8—C7	−0.47 (18)
C11—S2—C1—C2	−64.20 (9)	O1—N1—C8—C7	179.39 (12)
C11—S2—C1—S1	60.08 (7)	C7—C8—C9—C10	1.89 (18)
C13—S1—C1—C10	−173.77 (8)	N1—C8—C9—C10	−178.31 (11)
C13—S1—C1—C2	64.78 (10)	C8—C9—C10—C5	−1.31 (17)
C13—S1—C1—S2	−58.40 (8)	C8—C9—C10—C1	179.70 (11)
C10—C1—C2—C3	−45.80 (14)	C6—C5—C10—C9	0.02 (17)
S2—C1—C2—C3	−165.44 (8)	C4—C5—C10—C9	178.31 (11)
S1—C1—C2—C3	71.36 (12)	C6—C5—C10—C1	178.98 (11)
C1—C2—C3—C4	64.05 (14)	C4—C5—C10—C1	−2.73 (18)
C2—C3—C4—C5	−49.41 (15)	C2—C1—C10—C9	−165.62 (11)
C3—C4—C5—C6	−161.70 (12)	S2—C1—C10—C9	−44.44 (13)
C3—C4—C5—C10	20.01 (17)	S1—C1—C10—C9	72.78 (12)
C10—C5—C6—C7	0.79 (19)	C2—C1—C10—C5	15.41 (16)
C4—C5—C6—C7	−177.56 (12)	S2—C1—C10—C5	136.59 (10)
C5—C6—C7—C8	−0.27 (19)	S1—C1—C10—C5	−106.18 (11)
C6—C7—C8—C9	−1.09 (19)	C1—S2—C11—C12	−61.71 (11)
C6—C7—C8—N1	179.11 (11)	S2—C11—C12—C13	65.29 (14)
O2—N1—C8—C9	179.72 (12)	C11—C12—C13—S1	−62.18 (14)
O1—N1—C8—C9	−0.42 (17)	C1—S1—C13—C12	56.76 (11)

### Hydrogen-bond geometry (Å, °)

**Table:**

*D*—H···*A*	*D*—H	H···*A*	*D*···*A*	*D*—H···*A*
C13—H13A···O1^i^	0.97	2.58	3.4565 (18)	151
C7—H7A···Cg1^ii^	0.93	2.82	3.7164 (15)	162

Symmetry codes: (i) −*x*, −*y*+1/2, *z*−1/2; (ii) −*x*+3/4, *y*+3/4, *z*+1/4.
